# ε-Caprolactone-based solid polymer electrolytes for lithium-ion batteries: synthesis, electrochemical characterization and mechanical stabilization by block copolymerization†

**DOI:** 10.1039/c8ra00377g

**Published:** 2018-05-08

**Authors:** Andreas Bergfelt, Matthew J. Lacey, Jonas Hedman, Christofer Sångeland, Daniel Brandell, Tim Bowden

**Affiliations:** Department of Chemistry – Ångström Laboratory, Uppsala University Box 538 SE-751 21 Uppsala Sweden andreas.bergfelt@kemi.uu.se

## Abstract

In this work, three types of polymers based on ε-caprolactone have been synthesized: poly(ε-caprolactone), polystyrene-poly(ε-caprolactone), and polystyrene-poly(ε-caprolactone-*r*-trimethylene carbonate) (SCT), where the polystyrene block was introduced to improve the electrochemical and mechanical performance of the material. Solid polymer electrolytes (SPEs) were produced by blending the polymers with 10–40 wt% lithium bis(trifluoromethane)sulfonimide (LiTFSI). Battery devices were thereafter constructed to evaluate the cycling performance. The best performing battery half-cell utilized an SPE consisting of SCT and 17 wt% LiTFSI as both binder and electrolyte; a Li|SPE|LiFePO_4_ cell that cycled at 40 °C gave a discharge capacity of about 140 mA h g^−1^ at C/5 for 100 cycles, which was superior to the other investigated electrolytes. Dynamic mechanical analysis (DMA) showed that the storage modulus E’ was about 5 MPa for this electrolyte.

## Abstract 2

Three different polymers were synthesized and evaluated as solid polymer electrolytes: poly(ε-caprolactone) (PCL), polystyrene-poly(ε-caprolactone) (SC), and polystyrene-poly(ε-caprolactone-*r*-trimethylene carbonate) (SCT).

## Graphical abstract

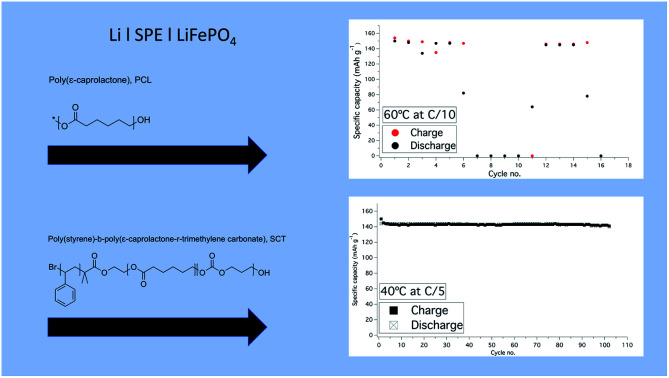

## Introduction

The field of solid polymer electrolytes (SPEs) for lithium ion battery devices has grown rapidly, especially since the electrification of the automotive industry has highlighted the demand for safer and cheaper batteries with higher energy densities.^1–5^ SPEs are good candidates to address these issues since they are less flammable and less reactive than liquid electrolytes with other battery components.^6^ Ideally, SPEs eliminate the need for an external separator, and their all-solid-state configuration can produce very mechanically robust battery cells. There is, however, normally a trade-off between mechanical and conductive properties in SPEs. As the mechanical properties improve, the ionic conductivity generally decreases since the ion transport is dependent on the segmental motion of the polymer chains.^7,8^

SPEs have long been dominated by poly(ethylene oxide) (PEO)-based materials. However, in recent years, a number of novel SPE chemistries and topologies have been evaluated, which has broadened the field to ion-coordinating units other than PEO-based systems. One such branch is based on polyesters, such as poly(ε-caprolactone) (PCL), and polycarbonates, *e.g.*, poly(trimethylene carbonate) (PTMC).^9–14^ PCL is a biocompatible polymer normally associated with excellent mechanical properties but with limited ionic conductivity since it has a high degree of crystallinity below 60 °C. Little work has been conducted on pure PCL SPEs, but Fonseca and Neves constructed a working LiNiCoO_2_|PCL|Li battery device based on PCL with 10 wt% LiClO_4_ that cycled for about 50 cycles. However, the charge and discharge capacity decreased drastically during cycling. AC impedance spectroscopy showed that the cell resistance was increasing with cycling, and the authors suspected that surface film formation on the electrodes was the reason for the fade in cycling performance.^15,16^ The LiClO_4_-based PCL electrolyte system was later investigated by Lin and Wu, where they studied the relationship between salt concentration, crystallinity and ionic conductivity.^17^

PTMC, in turn, is an aliphatic polycarbonate with a glass transition temperature (*T*_g_) of about −15 °C, that has the ability to form rubbery, amorphous, and transparent electrolytes after inclusion of salt. In two articles, Smith and Silva *et al.* pioneered the performance of high molecular weight (300 000 g mol^−1^) PTMC electrolytes, using lithium triflate, LiClO_4_, and LiBF_4_. The electrolytes were fully amorphous, but showed rather limited ionic conductivities.^18,19^ More lately, PTMC-based SPEs have proven to function in both lithium- and sodium-ion batteries, with good cycling stability that may be attributed to the high lithium ion transference number, T_+_, of 0.80 for a PTMC system with LiTFSI at 60 °C.^11,20,21^ This is much higher than values reported for poly(ethylene oxide) (PEO) electrolytes, which are typically reported to be in the range 0.1–0.3.^22–24^ Although being a very promising electrolyte host candidate, the ionic conductivity of PTMC at room temperature can be considered too low.

Mindemark *et al.* have therefore, in a series of papers, investigated random copolymers of trimethylene carbonate and ε-caprolactone with LiTFSI as electrolyte salt.^9,10,20^ For certain monomer ratios (80 : 20) and salt concentrations (36 wt%), the ionic conductivity reached 10^−4^ S cm^−1^ at room temperature, showing that there is a window within which suitable composition can enhance the overall ionic conductivity. Together with the high transference number for this system, it was possible to cycle lithium ion half-cell batteries at room temperature with a high capacity at a reasonable rate.^10,21^ The main drawback with the PCL : PTMC electrolyte is, however, that it is very soft and sticky. The mechanical properties were addressed in a later paper, where gamma irradiation was used to crosslink the electrolyte in an assembled device, but this strategy proved to have limited success since the material only showed a slight increase in storage modulus (G′) and the electrochemical performance was poor.^25^ Another option for improving the mechanical properties of an SPE material, without compromising the ionic conductivity, is to create a block copolymer (BCP), where one block creates ionic mobility and the other mechanical stability. Ideally, the BCPs should self-assemble into discrete microphase-separated domains, allowing for maximum gain from the individual components, *i.e.*, ionic conductivity and mechanical stability.^26–29^ It has been predicted that if a polymer coating with a high shear modulus (6 GPa) is applied to the anode, lithium dendrite formation can be suppressed, thus preventing a short circuit in the battery device.^30^

Regarding BCPs, several parameters must be addressed, which do not normally apply to SPEs. It is important not only to choose components that allow microphase separation to occur in order to maximize the individual block components, but also consider how changes in the monomer structure and polymer composition impact ionic conductivity, lithium ion transference number, electrochemical stability, solid electrolyte interphase (SEI) formation, and cycling performance – all key aspects for battery performance.^26,27,31,32^ In the context of BCPs for SPEs, much attention has been given to the polystyrene-poly(ethylene oxide) (PS-PEO) system that indeed combine both mechanical and electrochemical performance. But although the modulus is often high when the mechanically rigid phase is introduced, the ionic conductivity generally decreases to a level where plasticizing agents need to be added. Another solution is to increase the operating temperature to between 60 °C to 100 °C (not an obstacle for electric vehicles), but with the penalty that the mechanical properties will be lost and parasitic side reactions will start to be expressed, which will affect battery performance and life time.^28,33^

**Fig. 1:**
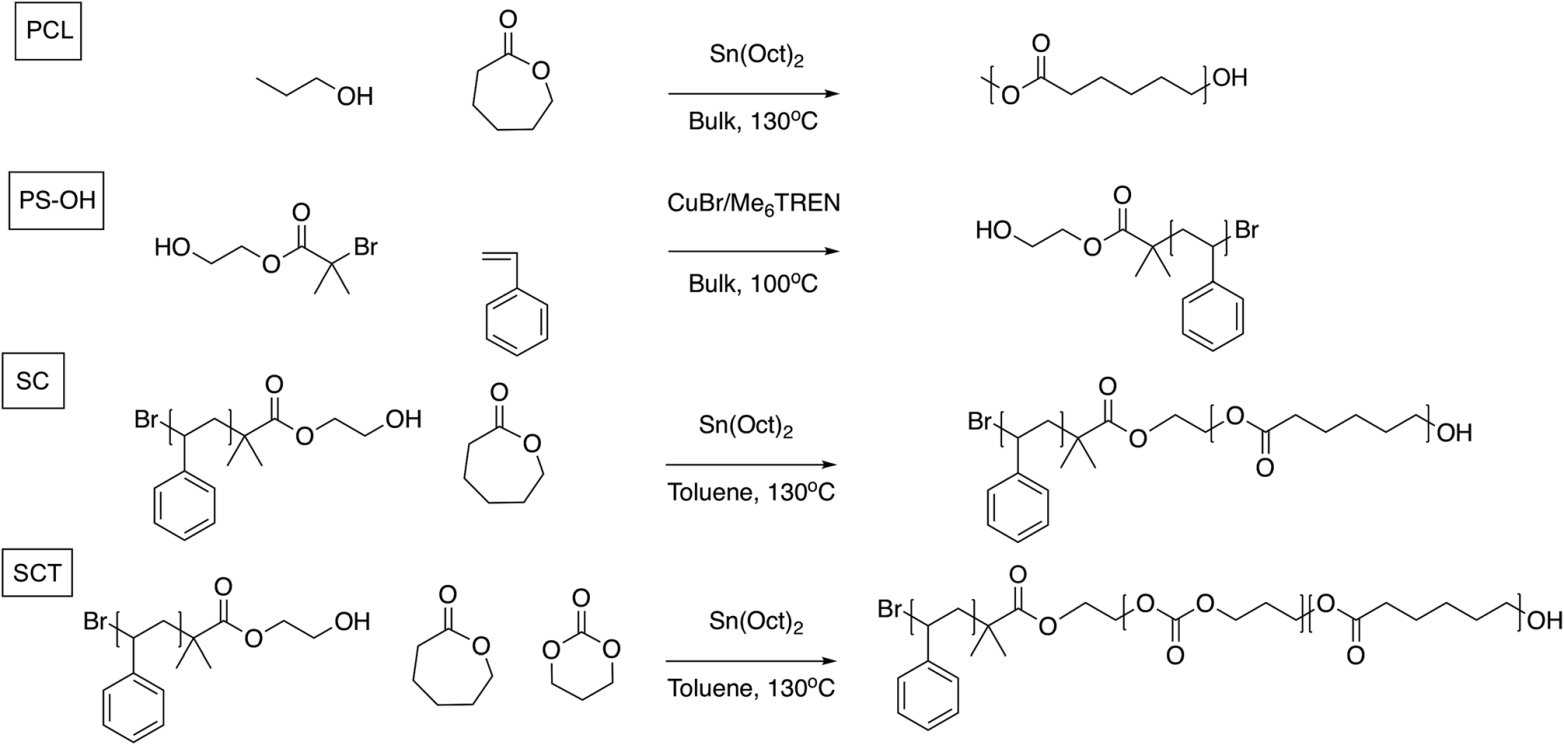
Reaction schemes for the synthesis of poly(ε-caprolactone) (PCL), polystyrene-poly(ε-caprolactone) (SC), and polystyrene-poly(ε-caprolactone-*r*-trimethylene carbonate) (SCT). PS-OH was used as a macroinitiator in the ring opening step to produce the SC and SCT block copolymers.

Inspired by the development of polyester and polycarbonate-based SPEs, and the block copolymer (BCP) design approach where mechanical and conductive properties could be combined *via* microphase separation, we here investigate and compare the electrochemical and battery cycling performance of three different polymers as SPEs: a poly(ε-caprolactone) (PCL) homopolymer, an AB diblock copolymer of polystyrene (PS) and poly(ε-caprolactone) (“styrene-caprolactone,” SC), and, finally, an AB diblock copolymer of PS coupled to a random copolymerized B-block of ε-caprolactone and trimethylene carbonate (“styrene-caprolactone-trimethylene carbonate,” SCT); see Fig. 1. By adding a styrene block to PCL the idea is to add mechanical properties to the SPE. In the SCT BCP the PCL block is randomly copolymerized with TMC in order lower the degree of crystallinity, in an attempt to improve the ionic conductivity. We also evaluate the SCT electrolyte as a cathode binder material since this should reduce any mass transport limitations in the cathode that could limit the overall performance of the battery device. As reported earlier, the advantage of these systems over PEO-based SPEs is that they combine favourable ionic conductivity with high transference numbers. To the best of our knowledge, this is the first non-ether-based PS block copolymer system to be investigated as a host material for an SPE.

## Experimental

### Materials

The materials used were the following: styrene-d_8_, tris[2-(dimethylamino)ethyl]amine (Me_6_TREN), 2-hydroxyethyl 2-bromoisobutyrate, CuBr_2_, stannous 2-ethylhexanoate, Al_2_O_3_ (Brockmann l, activated, basic), dry toluene, tetrahydrofuran (THF) (all from Sigma-Aldrich), ε-caprolactone (Sigma-Aldrich), trimethylene carbonate (Boehringer Ingelheim), dry tetrahydrofuran (Acros Organics), dry toluene (Acros Organics), methanol (Fisher Scientific), and CDCl_3_ (Larodan Fine Chemicals). Solvents were used without further purification. Lithium bis(trifluoromethane)sulfonimide (LiTFSI, Purolyte, Ferro Corporation) was dried at 120 °C for 24 h before use. Lithium iron phosphate (LFP, Phostech Lithium), super P carbon black (Erachem), poly(vinylidene fluoride) (PVdF, Kynar Flex 2801-00, Arkema), NMC cathode (MTI), graphite anode (MTI), and aluminium and copper foil were used as received.

### Poly(ε-caprolactone) (PCL) synthesis

ε-Caprolactone was dried with CaH_2_ overnight before being distilled and transferred to an argon-filled glove box; 2.42 mL of freshly distilled ε-caprolactone, 1 μL propanol, and 21.2 μL stannous 2-ethylhexanoate (0.1 M in toluene) catalyst were added to a pre-dried round bottom flask. This was capped with a silicone rubber septum and transferred to an oil bath set at 130 °C. The round bottom flask was removed after 24 h, and 20 mL of THF was added. The dissolved polymer was precipitated in 400 mL of methanol, collected, and dried in a vacuum oven at room temperature. *M*_n_ GPC: 24 392 g mol^−1^, PDI: 1.86, 1 mL min^−1^ GPC operating with THF at 35 °C using PMMA standards.

### Polystyrene macroinitiator (PS-OH) synthesis

Styrene was filtered through 10 mL basic Al_2_O_3_ to remove the inhibitor before polymerization. The monomer (40 mL styrene), initiator (0.1 mL, EBiB-OH), ligand (Me_6_TREN, 18.4 μL), and catalyst (1.5 mg, CuBr_2_) were added to a Schlenk flask. Oxygen was removed *via* three freeze–thaw cycles, using liquid nitrogen as a cooling agent. The flask was back-filled with argon, and 28 mg stannous 2-ethylhexanoate was added to start the reaction. The reaction was carried out in an oil bath set at 100 °C. The reaction was quenched after 7 h with 20 mL acetone, and the mixture was quickly filtered through a syringe filled with 30 mL basic Al_2_O_3_ capped with a 0.45 μm PVdF filter into a beaker with 400 mL methanol. The polymer was recovered *via* Büchi filtration and dried in a vacuum oven at room temperature. *M*_n_: 27 852 g mol^−1^, PDI: 1.13.

### Polystyrene-*b*-poly(ε-caprolactone) (SC) synthesis

The PS-OH macroinitiator (1 g) was transferred into an argon-filled glove box and mixed with 1 g of dry toluene in a pre-dried 20 mL vial. After the PS-OH was dissolved, 4 g ε-caprolactone and 24 μL (1 M in toluene) stannous 2-ethylhexanoate were added. The 20 mL vial was capped with a lid with aluminium inner foil before being transferred to a heating socket, where the polymerization was carried out at 130 °C. After 24 h, the vial was removed from the heating socket, and 10 mL of THF was added to dissolve the polymer. The solution was precipitated into a beaker with 400 mL of methanol, and the polymer was recovered *via* Büchi filtration and dried in a vacuum oven at room temperature. *M*_n_ GPC: 49 151 g mol^−1^, PDI: 1.43.

### Polystyrene-*b*-poly(ε-caprolactone-*r*-trimethylene carbonate) (SCT) synthesis

The PS-OH macroinitiator (1 g) was placed in an argon-filled glove box and mixed with 1 g of dry toluene in a pre-dried 20 mL vial. After the PS-OH was dissolved, 3.4 g ε-caprolactone, 0.57 g trimethylene carbonate, and 24 μL stannous 2-ethylhexanoate (1 M in toluene) were added. The 20 mL vial was capped with a lid with aluminium inner foil and transferred to a heating socket, where the polymerization was carried out 130 °C. After 24 h, the vial was removed from the heating socket, and *ca.* 10 mL of THF was added to dissolve the polymer. The solution was then precipitated into a beaker with 400 mL of methanol. The polymer was collected *via* Büchi filtration and dried in a vacuum oven at room temperature. *M*_n_ GPC: 51 210 g mol^−1^, PDI: 2.33.

### GPC

The GPC system was an Agilent 1260 Infinity instrument fitted with PolyPore columns and an RI detector. The mobile phase was THF (1 mL min^−1^) at 35 °C. PMMA standards were used to calibrate the system.

### NMR

A JEOL Eclipse+ 400 MHz spectrometer was used to record ^1^H-NMR spectra with CDCl_3_.

### Electrolyte film preparation

The polymers were mixed with dry THF and LiTFSI (*ca.* 100 mg of polymer and 2 mL THF with the corresponding amount of LiTFSI). The films were cast in Teflon moulds (20 mm in diameter), and the solvent was removed *via* controlled evaporation in a Büchi oven. The pressure was reduced to full vacuum (<1 mbar) over 20 h before heating at 60 °C at full vacuum for 40 h. The resulting films were 20 mm in diameter and *ca.* 0.2 mm in thickness. All work was performed in an argon-filled glove box.

### DSC

A TA instruments DSC Q2000 was used. The samples were hermetically sealed in aluminium pans in an argon-filled glove box to avoid moisture absorption. A cooling/heating/cooling/heating cycle was used from −80 °C to 160 °C with a ramping speed of 10 °C min^−1^ under a flow of N_2_. The second heating ramp was used to analyse the data.

### AC impedance

The ionic conductivity was measured by impedance spectroscopy using an SI 1260 Impedance Gain-Phase Analyser (Schlumberger) over the frequency range 1–10 MHz with the amplitude set to 10 mV. The electrolytes were sandwiched in Ni-plated stainless steel coin cells, CR2025, from Hohsen. The coin cells were heated to 90 °C and cooled to anneal the electrolyte with the electrode surfaces before the measurements were carried out from 30 °C to 90 °C. The samples were equilibrated at each temperature for *ca.* 20 min before a new recording was made. The resistance was evaluated with ZView (Scribner Associates) using a modified Debye circuit as a model.

### Cyclic voltammetry

Cyclic voltammetry was carried out on a VMP2 instrument (Bio-Logic). The cells were prepared with a 20 mm disc of polymer electrolyte sandwiched between a 14 mm lithium disc and an 18 mm stainless steel disc (Hohsen). The stack was sealed in a pouch bag. All work was carried out in an argon-filled glove box. The stainless steel discs were ultra-sonicated for 30 min in a 1 : 1 mixture of acetone and ethanol, and dried with a heat-gun before cell assembly.

### Transference number

Transference numbers were measured using an SP-240 instrument (Bio-Logic) connected to an oven operated at 60 °C for SC23 and 40 °C for SCT17. An electrolyte with a diameter of 20 mm was sandwiched between two 16 mm diameter lithium electrodes. The stack was then sealed in a pouch bag using copper current collectors. The cell was annealed at the OCV for 24 h before measuring the AC impedance. The cell was then allowed to rest for 1 h before applying a voltage step of 20 mV and recording the current *versus* time. After 60 h, the AC impedance was measured again. The AC impedance data was fitted with ZView, and the initial current was calculated by interpolation of the initial current.

### Battery cell preparation

LiFePO_4_ electrodes were prepared with a mixture of 75 wt% active material, 10 wt% carbon black, and 15 wt% PVdF on aluminium foil. The slurry was prepared with NMP as solvent. LiFePO_4_ electrodes with the SCT20 electrolyte as binder were prepared in a similar way, but with a mixture of 70 wt% LiFePO_4_, 15 wt% carbon black, and 15 wt% SCT17 electrolyte on aluminium foil. The slurry was prepared with THF as solvent. The electrodes were dried at 120 °C for 12 h before cell preparation. The electrolytes were cast on the cathodes and vacuum dried for 20 h before heating at 60 °C at full vacuum for 40 h and then assembled with lithium anodes and sealed in pouch cells. The full cells were prepared with commercial cathodes and anodes from MTI. The single-coated LiNiCoMnO_2_ cathode had a specific capacity of 155 mA h g^−1^ with a total thickness of 60 μm and an active material density of 121 g m^−2^. The anode was composite graphite on a copper foil containing SBR and CMC as binders, with a 50 μm coating thickness, an active material density of 60 g m^−2^, and a capacity of 330 mA h g^−1^. The electrodes were dried at 120 °C for 12 h before cell preparation. The SCT20 electrolyte was cast on the cathode and anode and vacuum dried for 20 h before heating at 60 °C at full vacuum for 40 h. Electrolyte-coated cathode and anode discs with a diameter of 16 mm were punched out, stacked on top of each other, and sealed in coin cells of stainless steel.

### Battery cycling

Galvanostatic cycling was carried out on a Digatron MBT-600 battery testing system at 40 °C between 2.7 and 4.2 V *vs.* Li^+^/Li for the LFP cells and 3.0 and 4.2 V at 40 °C for the NMC cells. The two-electrode setup for the intermittent current interruption technique was run on an MPG2 instrument (Bio-Logic).

### Rheology

Rheology measurements were performed on a Discovery Hybrid Rheometer (DHR 2, TA Instruments) with an 8 mm parallel plate geometry at 40 °C with an axial force of 5 N ± 0.5 N.

### Dynamic mechanical analysis

A DHR2 instrument with ETC was used in the DMA mode using film clamps. The measurements were performed at 1 Hz.

## Results and discussion

### Polymer synthesis

Three different polymers were synthesized: polycaprolactone (PCL), a diblock copolymer of PS and polycaprolactone (SC), and a diblock copolymer of PS coupled to a block of copolymerized ε-caprolactone and trimethylene carbonate (SCT). The synthesized PCL had molecular weights and polydispersity indices (PDI) that were expected for the chosen catalyst, stannous 2-ethylhexanoate; see Table 1. The diblock copolymer of PS and polycaprolactone (SC) was synthesized *via* a two-step synthesis. In the first step, a polystyrene macroinitiator (PS-OH) was synthesized by atom transfer radical polymerization (ATRP). The PS-OH macroinitiator was evaluated with both stannous 2-ethylhexanoate and Cu^0^ as reducing agents. Impurities and the catalyst complex that could cause electrochemical instability in the final battery device were removed by quenching the reaction mixture with acetone before filtration with basic Al_2_O_3_. After the filtration step the solution was fully transparent. Both reducing agents, stannous 2-ethylhexanoate and Cu^0^ foil, resulted in polymers with well-controlled molecular weights and narrow PDIs, indicating well-controlled polymerization conditions. However, stannous 2-ethylhexanoate was chosen because it gave higher reaction kinetics and it was possible to generate higher overall molecular weights of the final polymer compared to using Cu^0^ foil as the reducing agent. The ATRP initiator, 2-hydroxyethyl 2-bromoisobutyrate, was used since it can act as an initiator for the ring opening polymerization (ROP), *via* its hydroxyl end group. In the ROP, stannous 2-ethylhexanoate was used as catalyst, which gave an SC block copolymer with an average molecular weight (*M*_n_) of 49 151 g mol^−1^ and a PDI of 1.43.

**Table 1:** Molecular weights (*M*_n_) and polydispersity indices (PDI) determined by gel permeation chromatography (GPC) with THF at 35 °C

Entry	*M*_n_ [g mol^−1^]	PDI
PCL	24 392	1.86
PS-OH	27 852	1.13
SC	49 151	1.43
SCT	51 210	2.33

The same approach was used to synthesize the SCT block copolymer but with the addition of trimethylene carbonate in the ROP step. The PDI reached 2.33, implying a rather uncontrolled synthesis. Alternative catalytic systems were evaluated, such as DBU/TBD/TU in DCM, but did not result in a satisfactory copolymerization of ε-caprolactone and trimethylene carbonate to relatively high molecular weights. Analysis by ^1^H-NMR revealed that the SCT contained 69.8 wt% ε-caprolactone and 7.0 wt% trimethylene carbonate, resulting in a 90 : 10 molar ratio of ε-caprolactone to trimethylene carbonate; see Fig. SI-1–SI-3 in the ESI† for ^1^H-NMR spectra.

### Crystallinity and thermal properties

All electrolytes formed self-standing, rubbery, and non-sticky films. Differential scanning calorimetry (DSC) showed that the three salt-free polymers were highly crystalline, thus explaining their milky appearance; see Table 2 for DSC *T*_g_ values. For DSC curves, see Fig. SI-4–SI-6 in the ESI.† All DSC scans were run with a cooling/heating/cooling/heating sequence to explore the history of the samples, *i.e.*, microphase separations and shifts in glass transition (*T*_g_), crystallinity (*T*_c_), and melting temperature (*T*_m_). PCL0, SC0, and SCT0 (0 denotes no added salt; salt concentrations are then given as weight percentages) were all semicrystalline, but the general trend for all samples were that the addition of LiTFSI reduced the degree of crystallinity. The addition of salt had a non-linear effect on *T*_g_ for the PCL and SC electrolytes. This could be due to several competing parameters such as a lowering of the crystallinity, long-range ion interactions that lower the segmental motion of the polymer chains and a plasticizing effect of the anion. However, the *T*_g_ linearly increased with the addition of salt in the SCT series. Generally, it was also found that *T*_m_ and *T*_g_ shifted to lower temperatures on the second heating scan and that PCL27, SC23, and SC27 showed a melting peak on the first heating cycle but not on the second heating cycle. This phenomenon can be attributed to aging of the electrolytes, indicating that the electrolytes may show crystal growth over time. This could be potentially important from an application point of view since it could affect both battery and mechanical performance over time.^34–36^ With the addition of styrene and TMC the electrolytes became less crystalline, and since both PS and PTMC are amorphous as homopolymers, this indicates that ε-caprolactone is responsible for the crystallization, at least in the salt-free systems.^34^ Also, since SC and SCT are BCPs they have the possibility to microphase separate into mechanical and conductive nanostructured domains. However, a second *T*_g_, that of PS, was difficult to detect in both the SC and SCT samples, potentially indicating a lack of phase separation between the blocks. This might be due to the poorly controlled ROP step, generating BCPs with broad PDIs. Nanostructured phase separation is a delicate matter to control since it depends on several factors, such as the amount and type of salt, block length, monomer sequence in the blocks, the Flory–Huggins interaction parameter, annealing time of the samples and even which type of solvent that is used.

**Table 2:** DSC data for the three different electrolytes, including glass transition temperature (*T*_g_), crystallization temperature (*T*_c_), and crystal melting temperature (*T*_m_), from the second heating scan at 10 °C min^−1^

Entry	*T*_g_ [°C]	*T*_c_ [°C]	*T*_m_ [°C]
PCL0	−34.3	27.9	55.5
PCL9	−38.2	10.9	51.3
PCL17	−42.9	14.4	51.2
PCL23	−44.1	−1.5	45.0
PCL27	−40.2	—	—
SC0	−36.7	26.8	55.1
SC9	−37.5	21.0	53.4
SC17	−40.1	−8.5	45.6
SC23	−41.8	—	—
SC27	−35.4	—	—
SCT0	−63.0	−10.4	42.2
SCT9	−42.1	−14.4	36.7
SCT17	−42.9	—	—
SCT23	−37.1	—	—
SCT27	−24.8	—	—

### Ionic conductivity

**Fig. 2:**
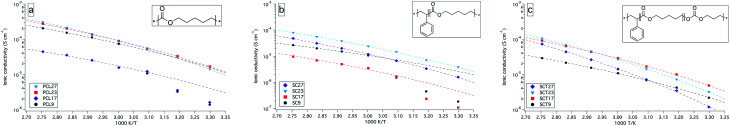
Ionic conductivity *versus* temperature plot (30 °C to 90 °C) for PCL, SC and SCT with 9, 17, 23, and 27 wt% LiTFSI. Dashed lines represent VTF fits.

The ionic conductivity for the electrolytes clearly show the effect of crystallinity on the ionic conductivity, which increases drastically when the crystalline content melts at about 50 °C to 60 °C; see Fig. 2. Generally, all the amorphous electrolytes show a Vogel–Tammann–Fulcher (VTF) behaviour, as shown in Fig. 2, which is expected for these types of systems where the ionic transport is supposed to be assisted by chain dynamics which is related to the *T*_g_ of the electrolytes. However, samples PCL17 and SC17 stand out from their series. It is hard to account for this, since their drop in conductivity is rather large when compared to their *T*_g_ values. However, it could be related to the samples thermal history, since the electrolytes were annealed at 90 °C for the AC impedance analysis and at 160 °C in the DSC analysis, which thus affects the thermal history and the degree of crystallinity.

The ionic conductivity does not linearly follow the amount of LiTFSI in the electrolytes but reaches a peak at certain concentrations, above which it decreases. This is generally observed for SPEs, and is due to the amount of dissociated charge carriers and long-range ion interactions, which affects the amount of free charge carriers and the segmental motion of the polymer chains.

The SC and SCT electrolytes showed a lower ionic conductivity than the PCL electrolytes, which probably is due to a lower volume of conductive phase, resulting from the added PS block. The addition of TMC in SCT did improve the conductivity slightly, compared to the SC electrolytes, following the results of Mindemark *et al.*^10^ The true nature of this improvement is probably the result of both a lower degree of crystallinity and a somewhat higher molecular flexibility, *i.e.*, a lower *T*_g_. But other factors should also be taken into consideration in future studies, such as balancing the dielectric constants of the coordination groups and the salt, and density of coordination groups. The lack of a second *T*_g_ for the SC and SCT electrolytes is as stated earlier a clear indication of that the BCP electrolytes are in the mixed state, which means that the mechanical and conductive properties blend. The result of this segmental mixing might affect the dissociation properties of the conductive block in a negative way and lower the amount of free charge carriers. It is expected that a fully microphase separated electrolyte with aligned domains should obtain a higher ionic conductivity than what is achieved here.

### Limited current fraction number and cyclic voltammetry

**Fig. 3:**
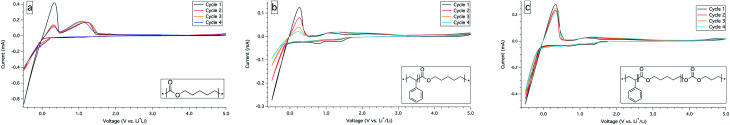
Cyclic voltammetry for electrolyte PCL23, SC23 and SCT17 with a cell configuration of stainless steel *versus* lithium recorded at 1 mV s^−1^ and at 60 °C for PCL23, SC23 and 40 °C for SCT17.

The electrolytes with the highest ionic conductivity in each series, PCL23, SC23, and SCT17, were evaluated electrochemically by cyclic voltammetry (CV) between 5 and −0.5 V *vs.* Li/Li^+^. The analysis showed that the electrolytes were stable at higher but not at lower voltages. For PCL23, there is a clear passivation on the first cycle – the current in the Li plating regime drops by a factor of 2 – indicating that any decomposition products formed is passivating the electrode. At about 1 V on the return scan, PCL23 shows a relatively high and broad current peak; see Fig. 3a. It is yet unclear what this current peak is an outcome of without any in-depth spectroscopic study of the degradation products formed at the electrode interface. However, a plausible explanation is that the ester groups in the polymers are redox active close to the lithium stripping and plating potential, forming degradation products. The same current peak in the Li plating regime is found for the SC23 and SCT17 electrolytes, however, not with the same passivation intensity in the first cycle and with possible redox active degradation products. The current flow at about 1–2 V is increasing slightly over time which indicates that degradation products is produced over time; see Fig. 3b and c.

The limiting current fraction numbers (F_+_) were estimated using the Bruce–Vincent method.^22^ The electrochemical instability of the PCL-based electrolytes made it impossible to obtain F_+_ for symmetrical lithium cells (Li|PCL23|Li) subjected to potentiostatic polarization, since they did not produce a steady-state current. However, the SC23 and SCT17 electrolytes produced steady-state currents, leading to a F_+_ of 0.56 and 0.68. See Fig. SI-7 to 10 in the ESI† for steady state current plots. This also indicates that although the SC and SCT electrolytes show several features in the CV scans, they are able to form a thermodynamically or kinetically stable interphase with the electrodes. The F_+_ values for the SC and SCT electrolytes are far higher than those usually reported for PEO systems, but are consistent with values published earlier for polycarbonate–polyester systems.^21^

### Battery cycling

A series of Li|PCL23|LiFePO_4_ half-cells were produced and cycled at 40 °C and 60 °C with different C-rates, but all cells failed after only a few cycles; the best performing cell managed only 16 cycles before failing, see Fig. SI-11 in the ESI.† The cells were annealed for 6 h at the operating temperature before cycling to maximize the interfacial contact between the electrolyte and the lithium anode. During this time, the open circuit voltage (OCV) fluctuated significantly, indicating reactions between the electrolyte and one of the electrodes, presumably the lithium anode, considering the results from the CV analysis.^37–39^ The fact that PCL is unstable *versus* lithium was also proven by the fact that a graphite|PCL23|LiFePO_4_ (with commercially available electrodes) full-cell device managed to cycle at 40 °C and at C/20 with *ca.* 60–100 mA h g^−1^ of LiFePO_4_ for over 50 cycles, see SI-12.†

**Fig. 4:**
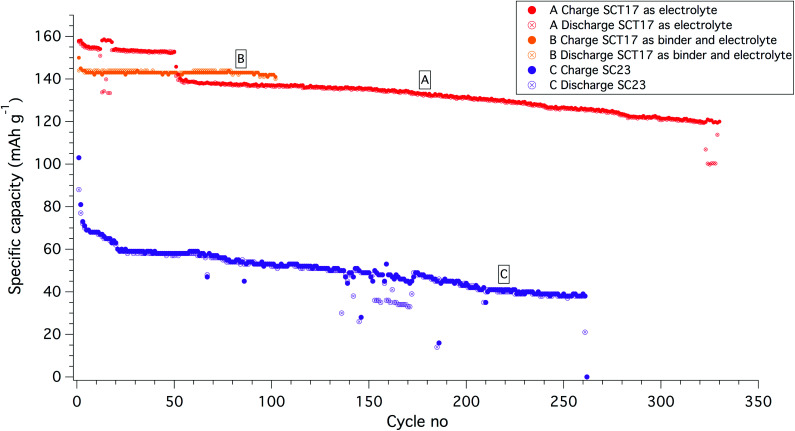
Cycling of Li|SPE|LiFePO_4_ half-cells. Battery A is cycling at C/10 with SCT17 as electrolyte. Battery B is utilizing SCT17 as a binder material in the cathode and as electrolyte, cycling at C/5. Both batteries were operated at 40 °C. Battery C is using SC23 as electrolyte, operating at C/10 and at 60 °C.

The Li|SC23|LiFePO_4_ cell, on the other hand, showed a flat OCV during the 6 h annealing at 60 °C, but upon cycling the capacity dropped significantly during the first two cycles. After this initial rapid drop, the capacity levelled out but continuously faded upon further cycling. Although showing a rapid decrease in capacity and a rather poor cycling stability the cell operated for over 250 cycles until it ultimately failed; see Fig. 4. This indicates that the addition of the PS block aids in stabilizing the SPE interphase with the lithium anode.

When cycling the Li|SCT17|LiFePO_4_ half-cells it was possible to decrease the temperature to 40 °C and at the same time cycle the cells at almost full capacity of the LiFePO_4_. SCT17 formed a smooth and hard electrolyte surface when solvent-casted onto the cathodes. The OCV was flat during the 6 h annealing step, indicating that the SCT electrolyte was sufficiently chemically and mechanically stable with the cell components. When comparing the cycling capability with the PCL and SC electrolytes, it can be concluded that the SCT17 electrolyte shows a superior cycling performance. A typical cycling behaviour of a SCT17 half-cell is shown in Fig. 4, battery A. The SCT-based cells are characterized by a stable cycling behaviour, and it should be noted that the cells tested do not fail as a result of “sudden death”, showing that the SCT electrolyte is indeed a highly reliable electrolyte material. An interesting phenomenon is that some of the SCT17-based half-cells showed a drop in capacity after about 40 to 60 cycles but then continued to cycle with no further interruptions. This coincided with a major increase in the cell overpotential, SI-13 in the ESI.† The reason for this increase in overpotential is hard to explain, but minor irreversible losses in the charge capacity did occur in association to this resistance increase.

**Fig. 5:**
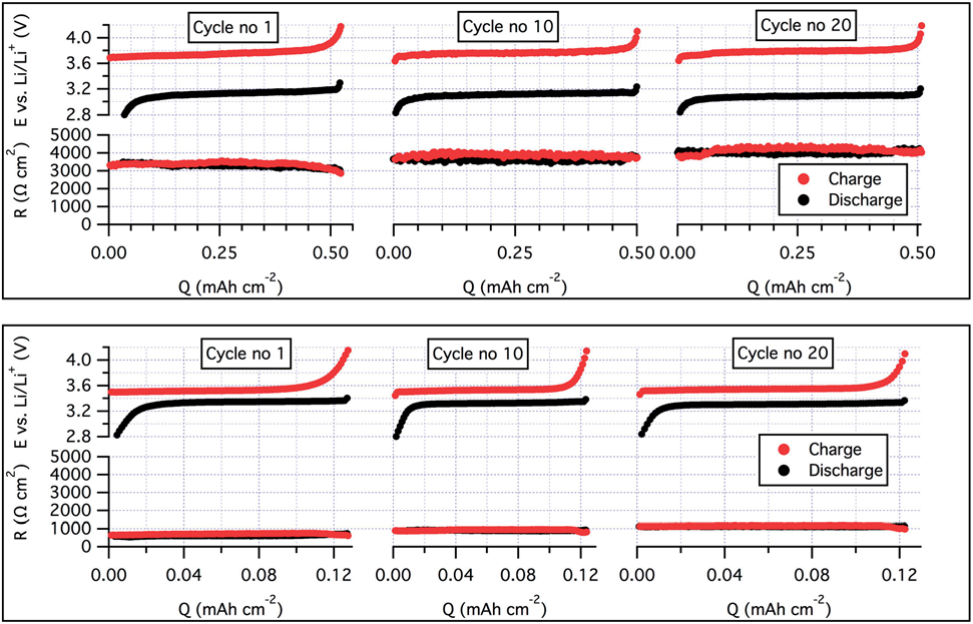
A two-electrode resistance analysis during cycles 1, 10, and 20 of a LiFePO_4_ half-cell cycling with SCT17 as electrolyte and PVdF as binder at 40 °C and at C/20 (upper image) and a LiFePO_4_ half-cell cycling with SCT17 as binder and electrolyte at 40 °C and at C/10 (lower image).

To ensure a good contact between the LiFePO_4_ (LFP) cathode and the SPE, the latter were solution casted onto the electrodes before a lithium disc was sandwiched on top of the electrolyte-coated cathode. To increase the mass transport of lithium ions through the cathode and generate good compatibility with the bulk electrolyte, a second batch of cathodes was produced, but with the SCT17 used both as a binder (instead of polyvinylidene difluoride; PVdF) and electrolyte.^40^ The cycling stability was greatly improved with this approach; see Fig. 4, battery B. With this approach it was possible to cycle the cell at C/5 and at almost full practical capacity of the LiFePO_4_ (145 mA h g^−1^, theoretical 170 mA h g^−1^). To understand the effect of using SCT17 as a binder in the cathode, an intermittent current interruption technique was applied to the Li|SCT17|LiFePO_4_ half-cell. In this experiment, the resistance was measured from short current interruptions during cycling of the cell.^41,42^ From Fig. 5, it is clear that the polarization and resistance during cycling are much higher in the cells using PVdF as binder material than in those using SCT17 as a binder. This means that the conventional solvent-casting of the SPE onto the cathodes is an insufficient fabrication method for SPE-based battery cells. Instead, the electrodes should be prepared with an electrolyte that to some degree already is present during electrode preparation to reduce resistance and polarization, which is a likely result of mass transport limitation in the inner parts of the cathode. Full-cells with the SCT17 electrolyte were also tested in coin cells, with commercially available LiFePO_4_ (LFP), LiNi_0.5_Mn_0.2_Co_0.3_O_2_ (NMC, 5 : 2 : 3), and graphite electrodes, in the configurations graphite|SCT17|LFP and graphite|SCT17|NMC. The coin cells displayed high polarization and comparatively low capacity, which also quickly faded upon cycling. The poor performance of these cells is probably due to relatively dense electrodes with a high mass loading, in combination with poor infiltration of the electrolyte during the solvent-casting procedure. Despite the high polarization and low capacity, it was nevertheless possible to cycle the NMC-based coin cells for more than 170 cycles, however with a low specific capacity utilization, probably as an outcome of poorly infiltrated electrodes; see SI-14 in the ESI† for cycling data.

### Mechanical testing

**Fig. 6:**
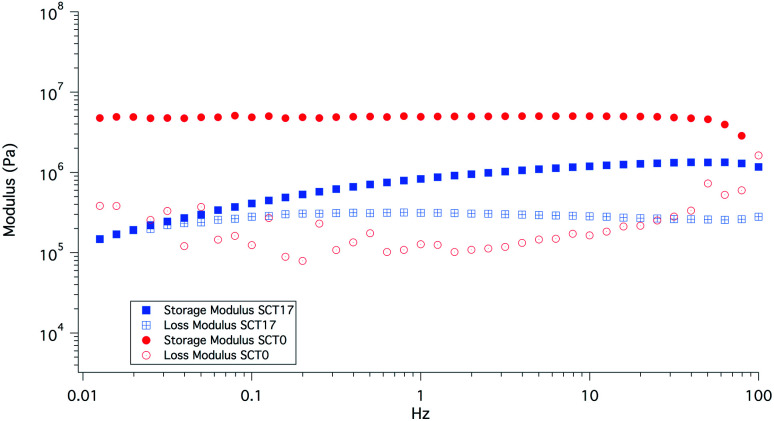
Comparison of storage and loss moduli of SCT0 and SCT27, measured at 40 °C.

The rheological properties of SCT17 and SCT0 were compared at different oscillation frequencies at 40 °C; see Fig. 6. It is clear that the addition of LiTFSI decreases the storage modulus G′, as a result the plasticizing effect of LiTFSI and the fact that SCT17 is amorphous. Where SCT0 shows no direct dependency on frequency, the modulus of SCT17 decreases continuously with frequency. At frequencies close to 0.01 Hz, G′ and G′′ for SCT17 almost coincide, indicating that the material may display viscous rather than rubbery properties at these frequencies.

**Fig. 7:**
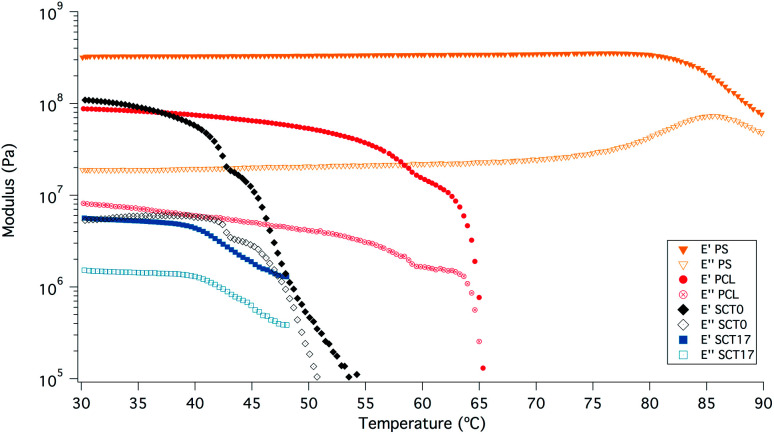
DMA results for samples SCT17, SCT0, PCL0, and polystyrene (PS), which was added as a reference material.

SCT17 was also analysed by dynamic mechanical analysis (DMA) to study the effect of temperature on the storage and loss moduli (E′ and E′′); see Fig. 7. SCT17 was compared with SCT0, PCL, and polystyrene (PS). PS showed the highest E′ and the modulus was steady up to 80 °C. PCL on the other hand showed a stable E′ until it reached its melting temperature of about 55 °C, and the modulus dropped significantly above 60–65 °C. SCT0 had a E′ between PCL and PS, however, as shown in Fig. 7, the drop in the modulus appeared at a lower temperature than that for both pure PS and PCL, which corresponds with the DSC value of the *T*_m_ of 42.2 °C, and the fact that the PCL block is randomly copolymerized with TMC. The addition of LiTFSI reduces E′, which is attributed to the plasticizing effect of the salt, resulting in an amorphous electrolyte.

These results are encouraging in terms of the mechanical stabilization provided by the block-copolymerization strategy, since SCT combines promising battery cycling capabilities with mechanical properties, comparable with many rigid systems.^26,28,29^

## Conclusions

Three polymer electrolyte series were synthesized and casted with LiTFSI, and their general electrochemical and battery device performances were evaluated. The first series utilized poly(ε-caprolactone) (PCL) as a host material, which showed the highest ionic conductivity of the three series. However, it was not possible to cycle a battery half-cell with the PCL electrolytes, which is believed to be because the electrolyte could not properly passivate the lithium anode. The second series was a styrene-caprolactone (SC) block copolymer. The battery devices that were assembled with this electrolyte could be cycled at 60 °C, although with a low capacity. The cycling data clearly indicated an initial reaction with the lithium anode that lowered the cycling capacity to about 100 mA h g^−1^ of LiFePO_4_. Nevertheless, the addition of the PS block resulted in a much more stable cycling behaviour, and it was possible to cycle a cell for about 250 cycles. The third series, a diblock copolymer of styrene-*b*-caprolactone-*r*-trimethylene carbonate (SCT), was synthesized so that the B-block was a copolymer of ε-caprolactone and trimethylene carbonate. By adding PS the mechanical properties increased, and by adding TMC the amount of crystallinity decreased. Although the addition of TMC only slightly improved the ionic conductivity, the cycling stability of the half-cells was greatly improved. The cycling data showed a superior steady cycling behaviour compared to the PCL and SC electrolytes, with half-cells cycling at almost full capacity at 40 °C and at C/5. Moreover, the battery cycling performance was improved by incorporating the electrolyte as a cathode binder material, thus reducing mass transport limitations in the cathode and lowering the overall cell polarization and resistance.

This indicates that SPE-based cells should have a different construction design compared to liquid-based cells. This study also highlights the use of PCL as a SPE host material and that PCL show promising properties, but also clear limitations due to a poorly stabilized interphase with lithium. However, when using a block copolymer approach we show that it is possible to stabilize this interphase, and at the same time add mechanical properties to the electrolyte. Using BCPs is a promising platform approach for future systematic studies, making is possible to change the type and composition of monomers and thus alter the microphase separation, electrochemical performance and finally the battery performance. Also, the electrochemical stability of the PCL homopolymer should be studied, but to do so a new type of sample preparation and post mortem analysis needs to be developed in order to properly analyse any degradation products formed at the electrode interphase.

## Conflicts of interest

There are no conflicts of interest to declare.

## Supplementary Material

RA-008-C8RA00377G-s001
