# Supplementary material for: ε-Caprolactone-based solid polymer electrolytes for lithium-ion batteries: synthesis, electrochemical characterization and mechanical stabilization by block copolymerization
Source: RSC Adv. 2018 May 8;8(30):16716–25. doi: 10.1039/c8ra00377g (PMC9082565; doi:10.1039/c8ra00377g)
Supplement: RA-008-C8RA00377G-s001 [file RA-008-C8RA00377G-s001.pdf]

Electronic Supplementary Material (ESI) for RSC Advances.
This journal is © The Royal Society of Chemistry 2018

ε-Caprolactone-based Solid Polymer Electrolytes for Lithium-Ion Batteries: Synthesis, Electrochemical Characterization and Mechanical Stabilization by Block Copolymerization

Andreas Bergfelt, Matthew J. Lacey, Jonas Hedman, Christofer Sångeland, Daniel Brandell, and Tim Bowden*

Department of Chemistry - Ångström Laboratory, Uppsala University, Box 538, SE-751 21, Uppsala, Sweden

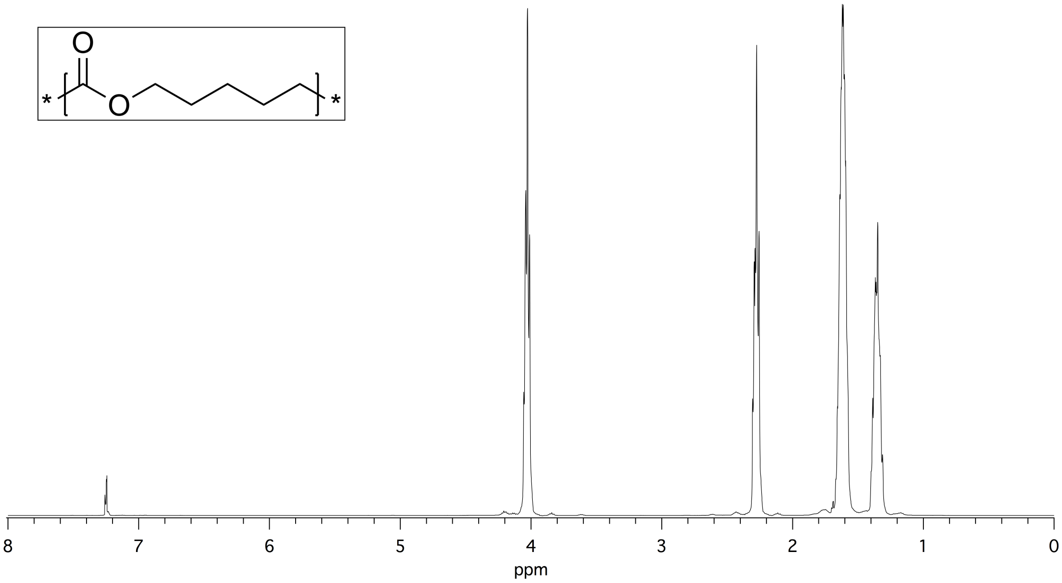

SI-1. ^{1}H-NMR spectra of PCL recorded in $CDCl_3$ that appears at 7.3 ppm.

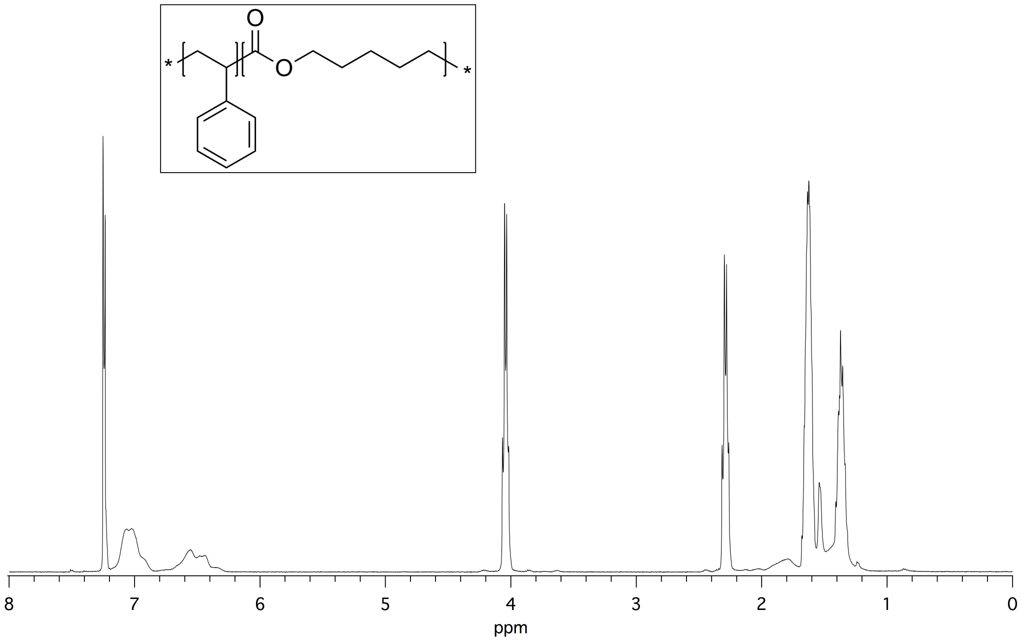

SI-2. ¹H-NMR spectra of the SC diblock copolymer. The solvent $CDCl_3$ appears at 7.3 ppm.

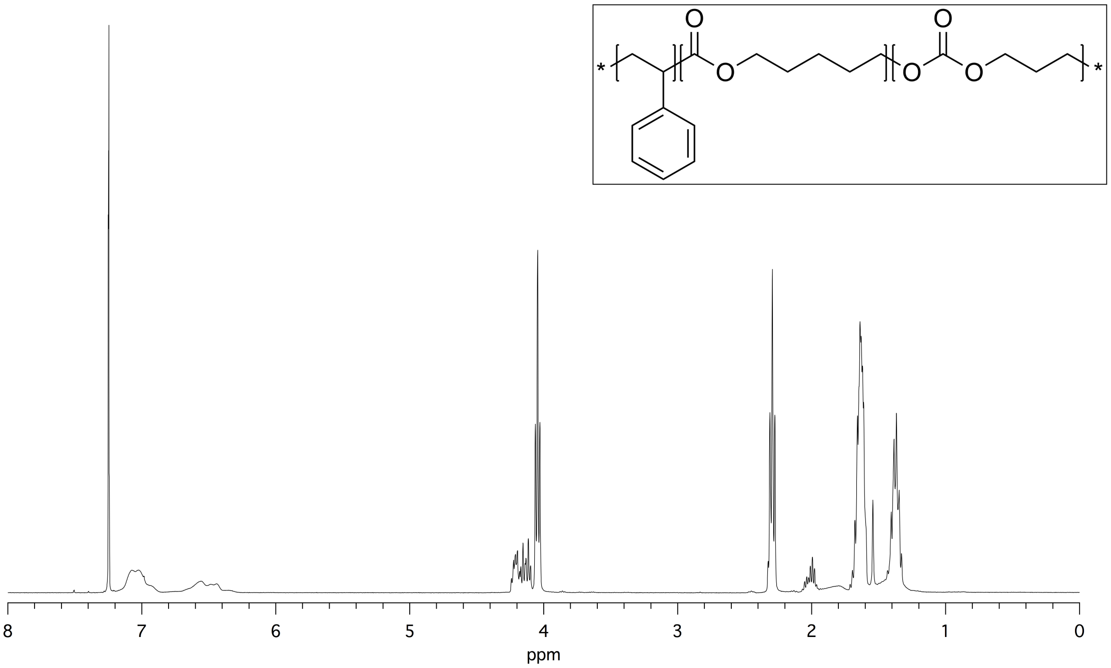

SI-3. ¹H-NMR spectra of the SCT diblock copolymer showing the characteristic PS region at about 7 ppm, PCL at 4 ppm, and PTMC at about 4.1 ppm. $CDCl_3$ appears at 7.3 ppm.

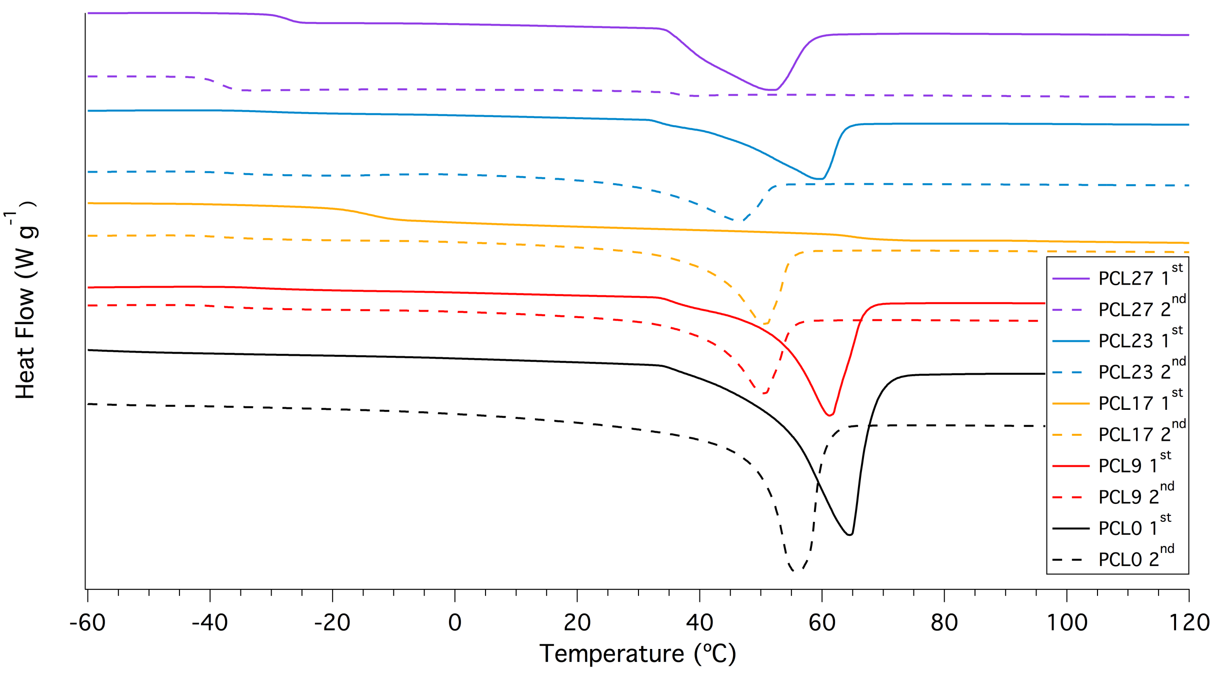

SI-4. DSC curves for PCL. The solid lines represent the first heating scan, and the dashed lines represent the second heating scan. The curves are shifted vertically for the sake of clarity.

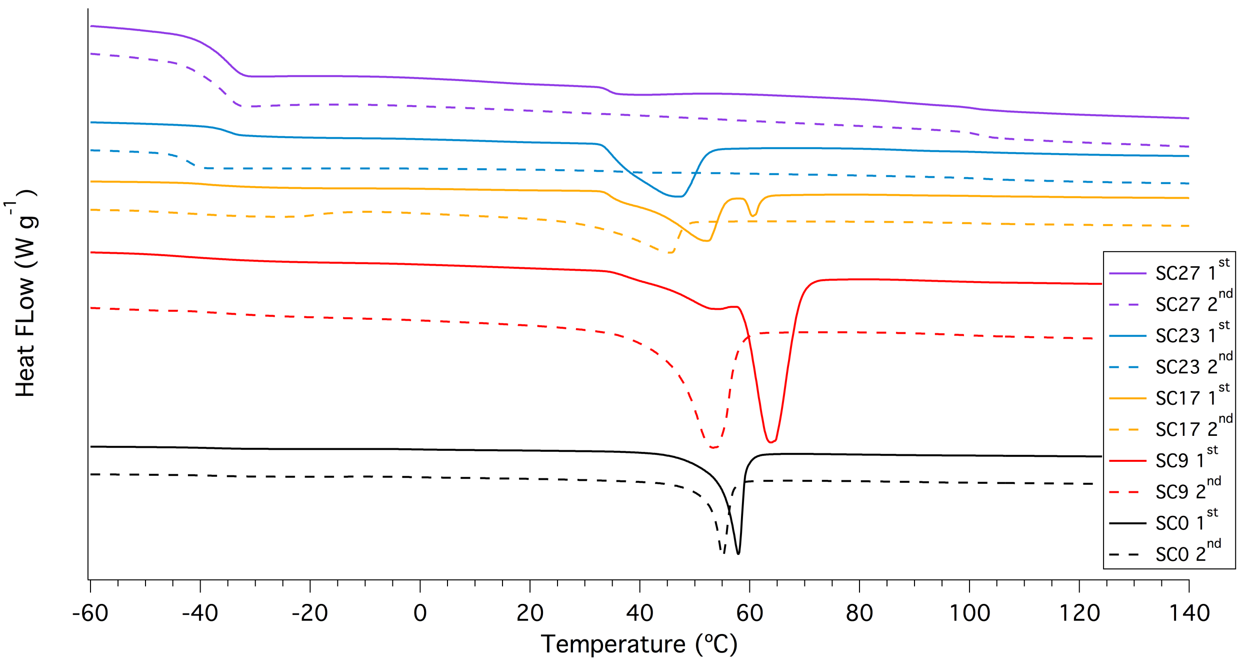

SI-5. DSC curves for SC. The solid lines represent the first heating scan, and the dashed lines represent the second heating scan. The curves are shifted vertically for the sake of clarity.

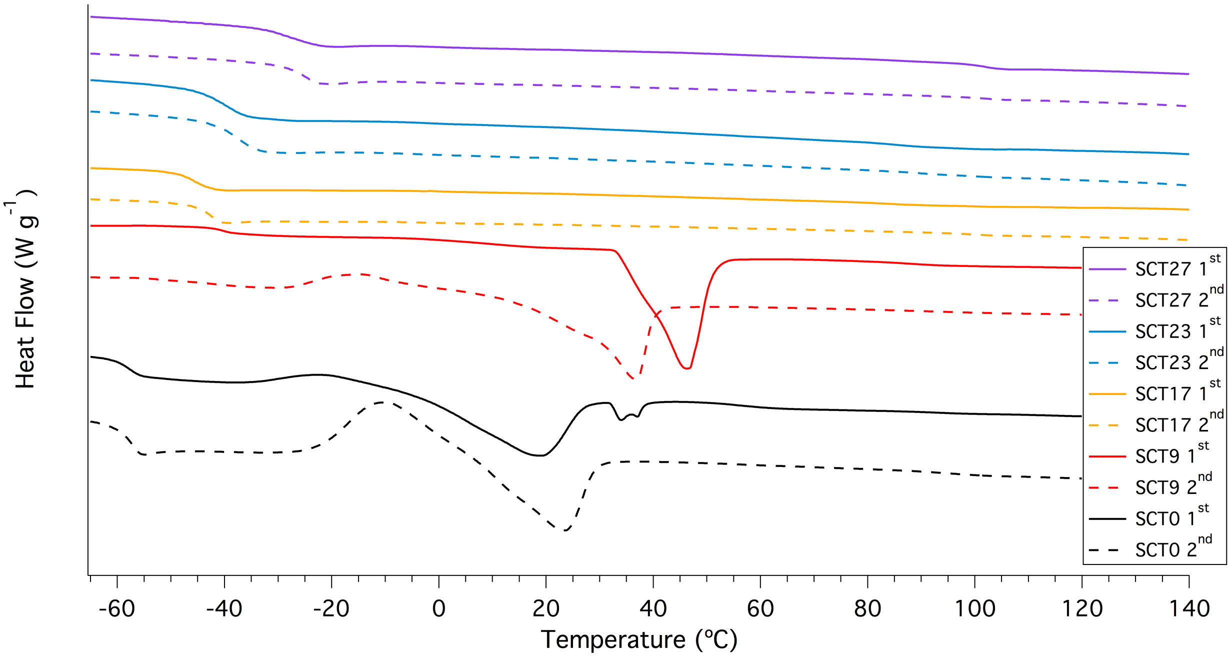

SI-6. DSC curves for SCT. The solid lines represent the first heating scan, and the dashed lines represent the second heating scan. The curves are shifted vertically for the sake of clarity.

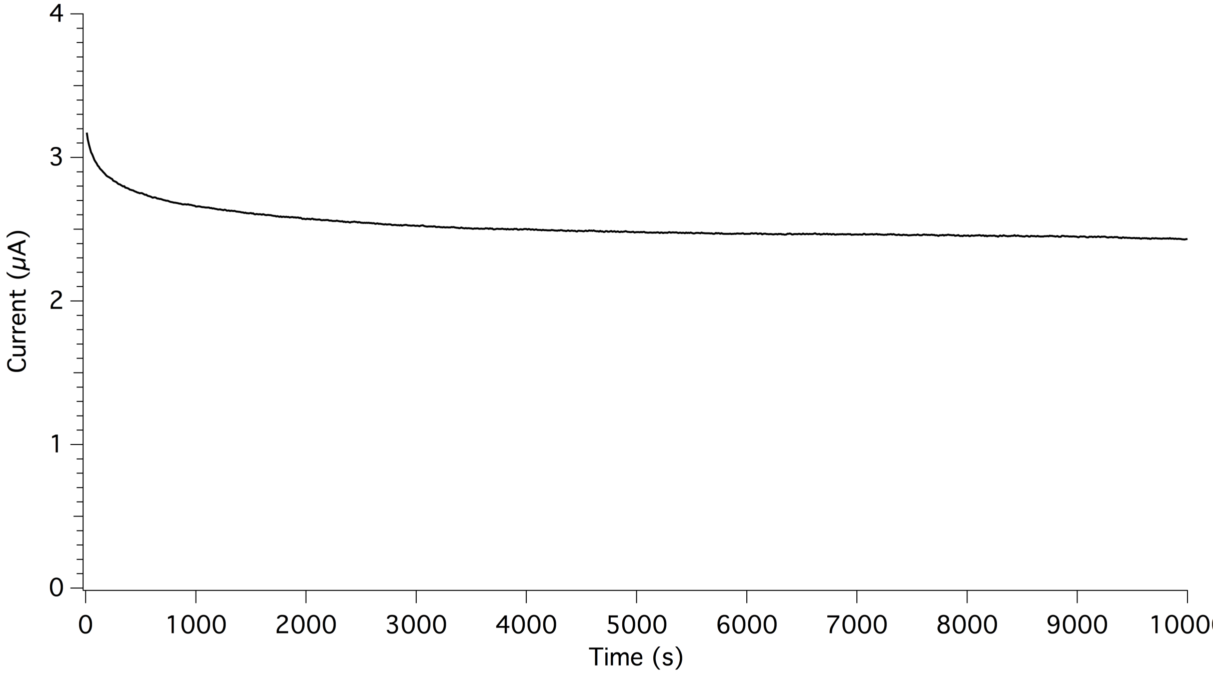

SI-7. Chronoamperogram of SC23, measured in a symmetrical Li | SC23 | Li cell at 60°C, with a voltage step of 20 mV.

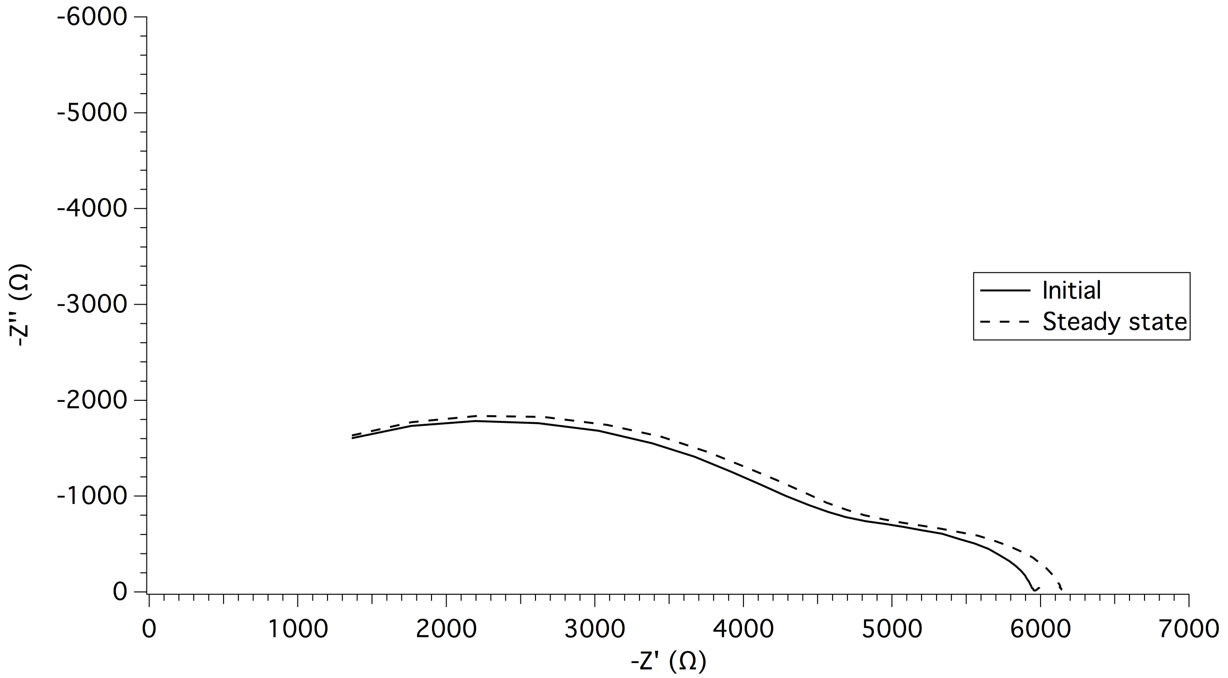

SI-8. Initial impedance before voltage step, and steady state impedance data for a symmetrical Li | SC23 | Li cell at 60°C.

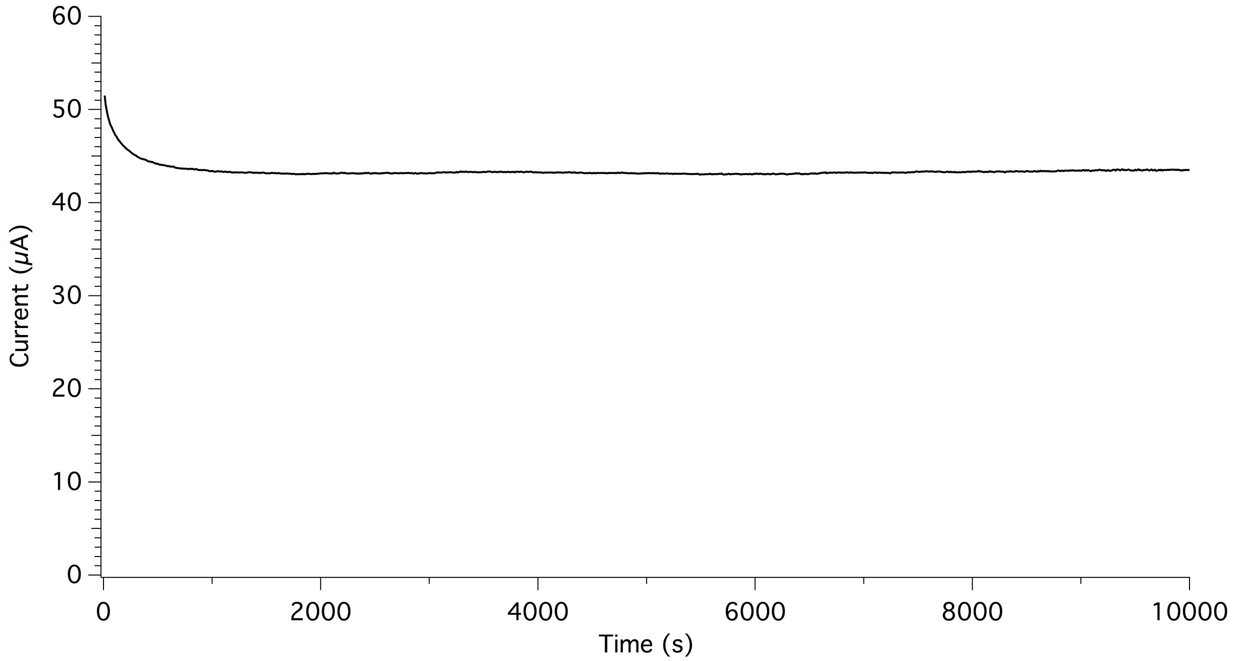

SI-9. Chronoamperogram of SCT17, measured in a symmetrical Li | SCT17 | Li cell at 40°C, with a voltage step of 20 mV.

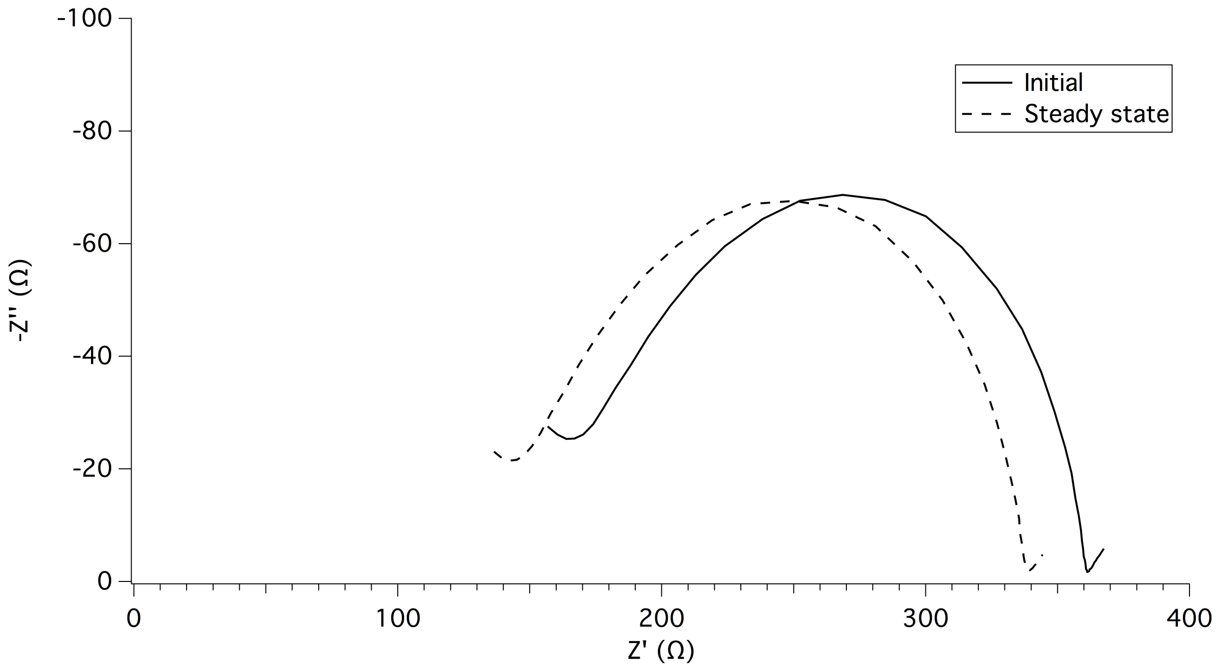

SI-10. Initial impedance before voltage step, and steady state impedance data for a symmetrical Li | SCT17 | Li cell at 40°C.

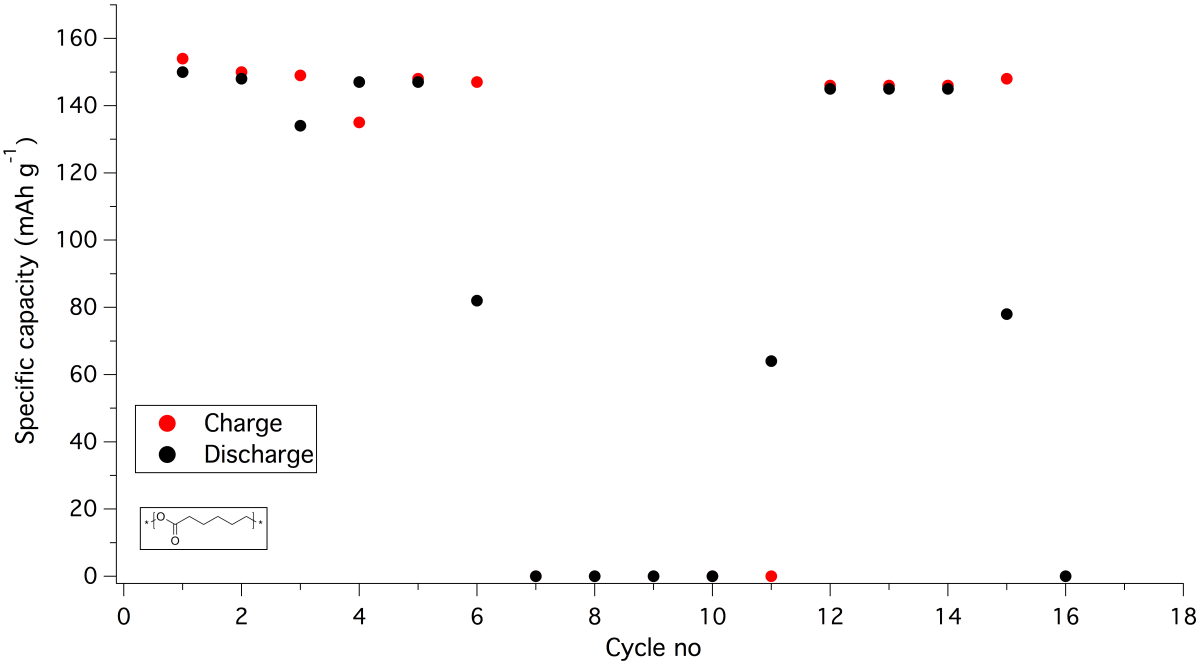

SI-11. Cycling of a Li | PCL23 | $LiFePO_4$ half-cell operating at 60°C. No PCL-based device operated for more than about 15 cycles before failing.

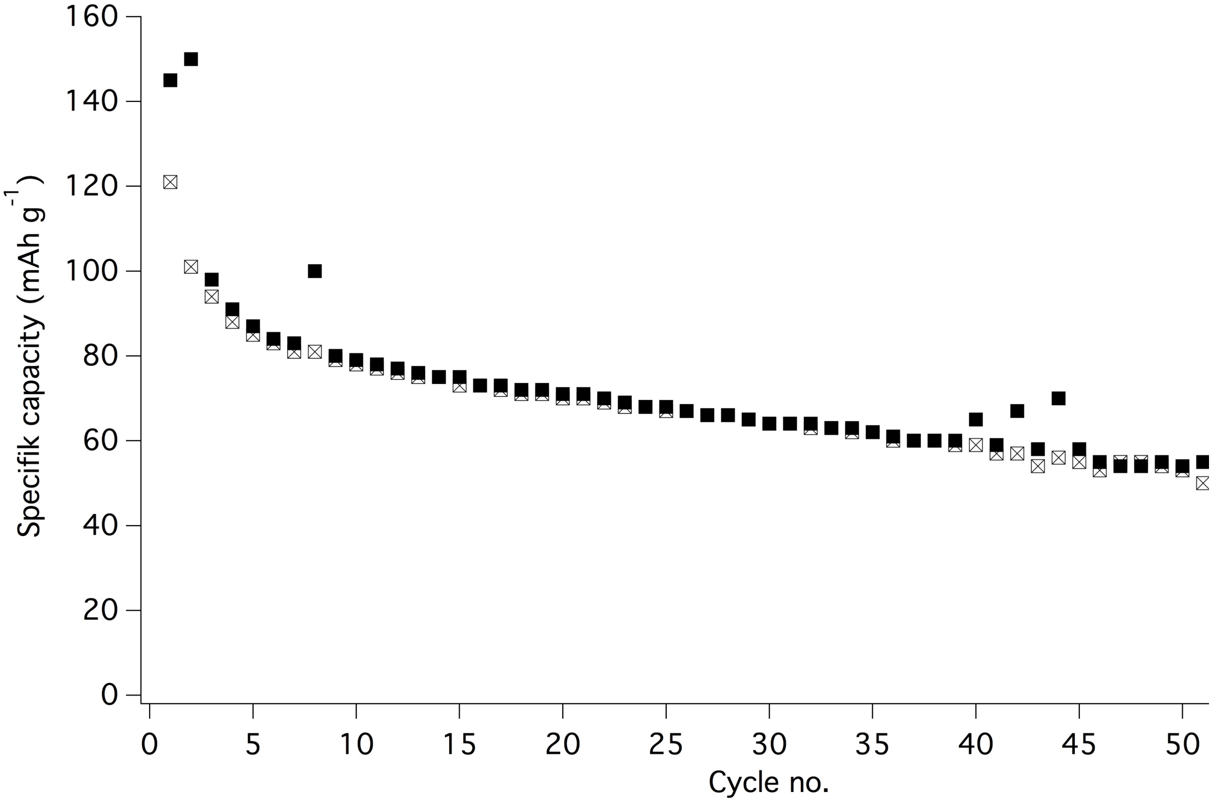

SI-12. Graphite | PCL29 | $LiFePO_4$ coin-cell operating at 40°C and at C/20.

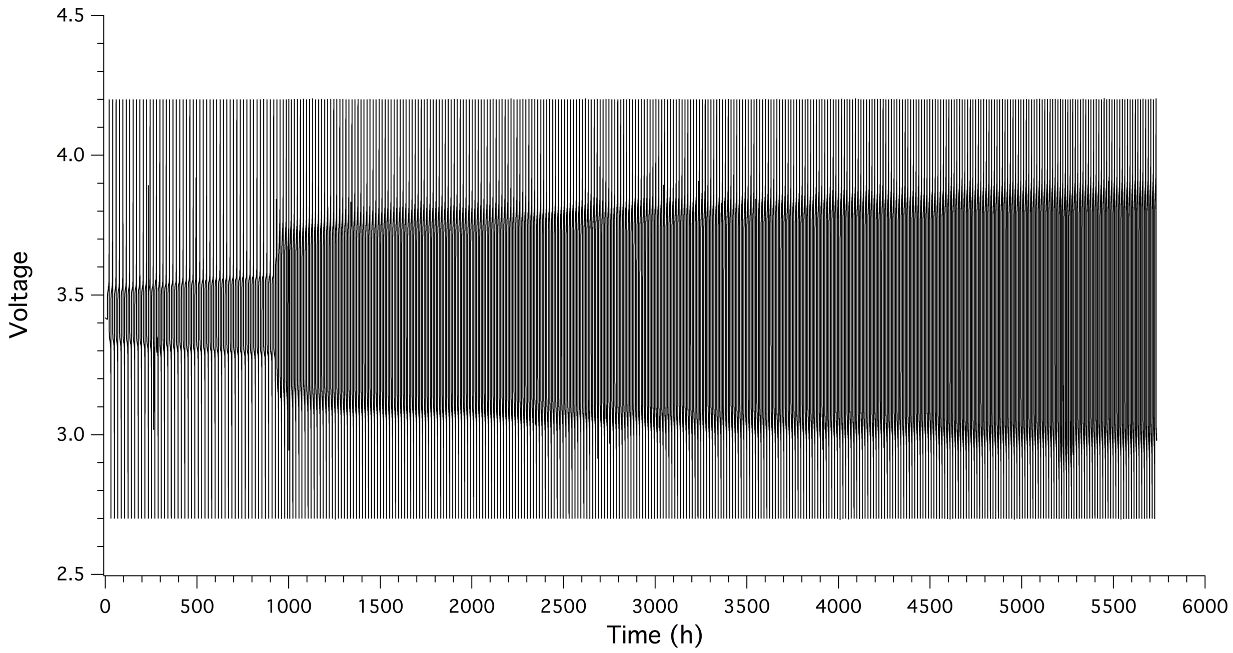

SI-13. Voltage profile for Li | SCT | $LiFePO_4$ half-cell operating at 40°C and at C/10.

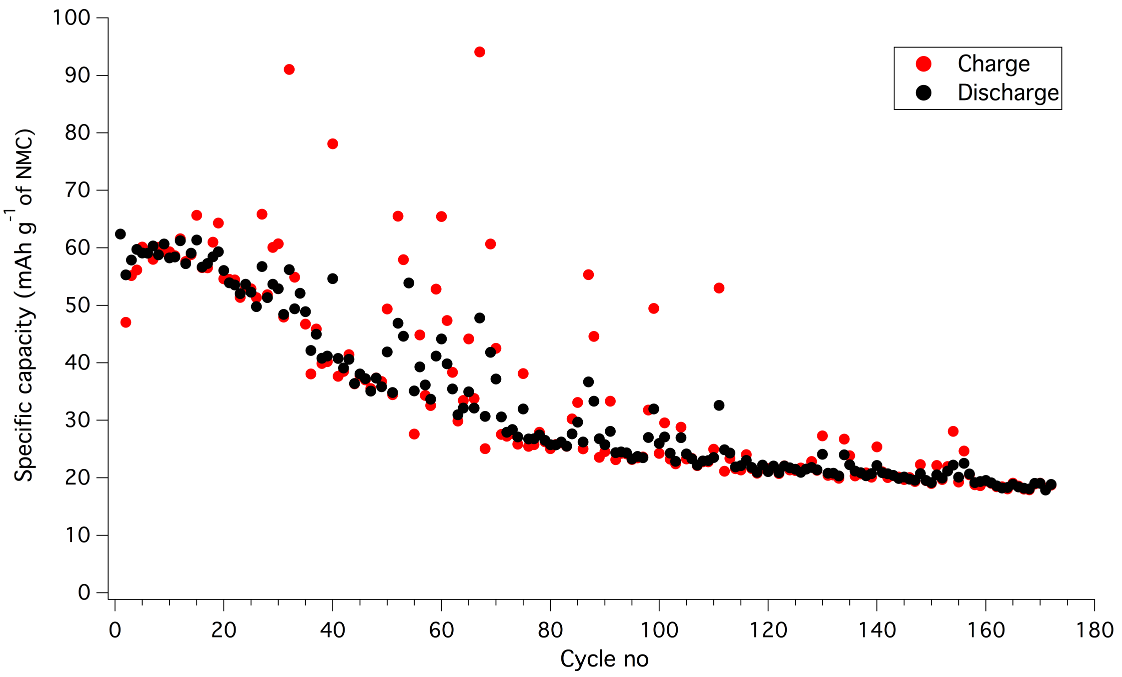

SI-14. Cycling of a coin cell device cycling 40°C and at C/10 with the configuration graphite | SCT17 | NMC. SCT17 was solvent cast onto the electrodes before they were sandwiched together and annealed at 40°C for 6 h before cycling.
